# GDF-15, iron, and inflammation in early chronic kidney disease among elderly patients

**DOI:** 10.1007/s11255-016-1278-z

**Published:** 2016-04-04

**Authors:** Ewelina Lukaszyk, Mateusz Lukaszyk, Ewa Koc-Zorawska, Anna Bodzenta-Lukaszyk, Jolanta Malyszko

**Affiliations:** 2nd Department of Nephrology and Hypertension with Dialysis Unit, Medical University of Bialystok, Marii Skłodowskiej – Curie 24a, 15-276 Białystok, Poland; Department of Allergy and Internal Medicine, Medical University of Bialystok, Bialystok, Poland

**Keywords:** GDF-15, Chronic kidney disease, Iron, Inflammation, Hepcidin

## Abstract

**Purpose:**

The aim of the study was to assess GDF-15 and iron status in patients in early stages of chronic kidney disease with a particular emphasis on elderly population in association of classic iron status parameters with novel iron homeostasis biomarkers and inflammatory parameters.

**Methods:**

Serum concentrations of GDF-15, iron (Fe), transferrin saturation, soluble transferrin receptor (sTfR), hepcidin, high-sensitive C-reactive protein (hsCRP), interleukin 6 (IL-6), and hemoglobin were measured in 56 patients ≥65 years of age and in 31 <65 years of age.

**Results:**

In patients ≥65 years, serum concentrations of GDF-15 and hsCRP were significantly higher in comparison with younger group. There was no statistically significant difference in hemoglobin, iron, hepcidin, and sTfR concentration between the two studied groups. In both groups GDF-15 was significantly correlated with hemoglobin, eGFR, hsCRP, and IL-6. GDF-15 was significantly higher in patients with anemia in comparison with their non-anemic counterparts. In multivariate analysis, hemoglobin was found to be a predictor of log GDF-15 (beta value = 0.36, *P* = 0.006, *R*^2^ = 37 %).

**Conclusions:**

An intricate interplay between renal function, anemia, and GDF-15 concentrations awaits further studies, as there are few data regarding pathophysiological role of GDF-15 in diabetes, kidney disease, and other comorbidities. Further understanding regarding the signaling pathways of GDF-15 may help to discover next pieces in the exciting puzzle of GDF-15. Finally, as studies dealing with normal level of GDF-15 in the healthy aged are lacking, it is possible that the higher values of GDF-15 values found in the present study represent values of GDF-15 according to age more than CKD levels.

## Introduction

In the Baltimore Longitudinal study, 254 healthy participants (without CKD, hypertension, not treated with diuretics) had periodical assessment of creatinine clearance in the period between 1958 and 1981 [1]. The mean decrease in creatinine clearance was 0.75 ml/min/year and was greater in patients who developed hypertension later during observation. In one in three patients, there was no fall in creatinine clearance. In NHANES III, an eGFR (MDRD) below 60 ml/min was found in 11 % of subjects over 65 years without concomitant diabetes and hypertension, while this was found in only 0.1 and 1.2 % subjects aged 20–39 and 40–59 years, respectively [2]. In our previous study, we found a very high prevalence of CKD up to 61 %, on the basis of estimated GFR/creatinine clearance, in elderly patients with coronary artery disease and normal serum creatinine level undergoing PCI [3].

One of the key pathomechanisms responsible for the progression of CKD is subclinical inflammation, which may be the result of decreasing clearance of uremic cytokines and oxidative stress. In some patients the cause of inflammation may be associated with concomitant diseases, including heart failure, atherosclerosis, or pulmonary disorders.

In most patients with CKD, there is a disruption in iron metabolism due to inflammation that may lead to anemia of inflammation (AI). However, iron deficiency (ID) is not necessarily linked to the occurrence of anemia. Elderly population is particularly vulnerable for the development of ID and AI.

The key element involved in iron metabolism is hepcidin; however, studies on new indices of iron status are in progress. Hepcidin is an acute phase protein that is synthesized to restrict the body’s iron stores and to prevent iron being requisitioned by invading microorganisms. One of the proteins involved in hepcidin-25 regulation and metabolism is growth differentiation factor-15 (GDF-15). GDF-15 is a member of transforming factor-β superfamily, which is secreted by erythroblasts as they mature. GDF-15 concentrations are elevated in the situations associated with ineffective erythropoiesis, such as thalassemia syndromes or sickle cell syndromes as well as other conditions like inflammation, acute injury, or cancer [4]. Moreover, GDF-15 is known as a cardiovascular risk marker independently of traditional risk factors such as CRP.

It has been shown that high concentration of GDF-15 is responsible for the reduced synthesis of hepcidin [5]. Therefore, GDF-15 is potentially involved in iron homeostasis. In patients with chronic kidney disease, serum concentration of GDF-15 is elevated [6]. GDF-15 is also presumed as an anti-inflammatory cytokine.

The aim of the study was to assess the GDF-15 and iron status in patients with early stages of chronic kidney disease with particularly emphasis on elderly population. Additionally, the association of classic iron status parameters with novel iron homeostasis biomarkers and inflammatory parameters was investigated.

## Patients and methods

Patients with established diagnosis of CKD (*n* = 87) were enrolled to the study. The inclusion criteria were: >18 years of age, an estimated glomerular filtration rate (eGFR) ≤60/ml/min/1.75 m^2^ or presence of hematuria or proteinuria for ≥3 months. Exclusion criteria included: clinically active acute inflammation (screening C-reactive protein, CRP >10 mg/dl) or thrombosis; active oncological disease; acute cardiovascular complication (including uncontrolled hypertension, acute coronary syndrome, acute heart failure); any anemia and/or iron deficiency treatment; blood transfusions within 3 months preceding the study; immunosuppressive therapy. The study protocol was approved by the Medical University Ethics Committee. All patients were fully informed about the study and gave their consent.

Medical history (including demographic characteristics as well as current pharmacotherapy) and blood samples were collected from all the subjects at the time of enrollment in the morning after an overnight rest. Diagnosis of CKD in >65 years was based the holistic diagnostic approach including nephrological and comprehensive geriatric evaluation of the patients, including mental status, not exclusively on the eGFR critical value of >60 ml/min for more than 3 months. We also ruled pout the mental impairment as GDF-15 have been associated with cognitive impairment, more common in the elderly [7]. Hematological measurements were made using fresh venous blood with EDTA and clotted blood. The plasma and serum were centrifuging and frozen at −70 °C until further laboratory analysis. Patients were divided into two groups: ≥65 (*n* = 56) and <65 (*n* = 31). Healthy volunteers (*n* = 20) were included in the study to obtain normal ranges for studied parameters.

Serum hemoglobin, creatinine, iron, total iron-binding capacity (TIBC), and ferritin levels were obtained using standard laboratory methods (automated system) in certified local central laboratory. Transferrin saturation (TSAT) was calculated as the ratio of serum iron and TIBC and expressed as a percentage. eGFR was calculated from chronic kidney disease epidemiology collaboration (CKD-EPI) equation according to the latest KDIGO guidelines [8].

Commercially available kits were used to measure: growth differentiation factor 15—GDF-15 (Quantikine ELISA Kit, R&D Systems, Minneapolis, USA), hepcidin-25 (EIA Kit, Peninsula Laboratories, LLC, Bachem Group, USA), soluble transferrin receptor—sTfR (Quantikine IVD ELISA Kit, R&D Systems, Minneapolis, USA), interleukin 6—IL-6 (Quantikine ELISA Kit, R&D Systems, Minneapolis, USA), and high sensitivity C-reactive protein—hsCRP (CRP Elisa Kit- LDN Labor Diagnostika Nord GmbH&Co KG, Nordhorn, Germany).

Data with normal distribution were reported as mean ± standard deviation and non-normally distributed as median and interquartile range. Variables with skewed distribution were log (ln)-transformed before further statistical analysis. Analyses of the correlation of each parameter were performed using Pearson or Spearman correlation coefficients. The multiple regression analysis was used to determine independent factors affecting the dependent variables. Factors showing linear correlation with GDF-15 (*P* < 0.05) were included in the analysis. All statistical analyses were performed using Statistica 10.0 (StatSoft, Tulsa, OK, USA) computer software.

## Results

**Fig. 1:**
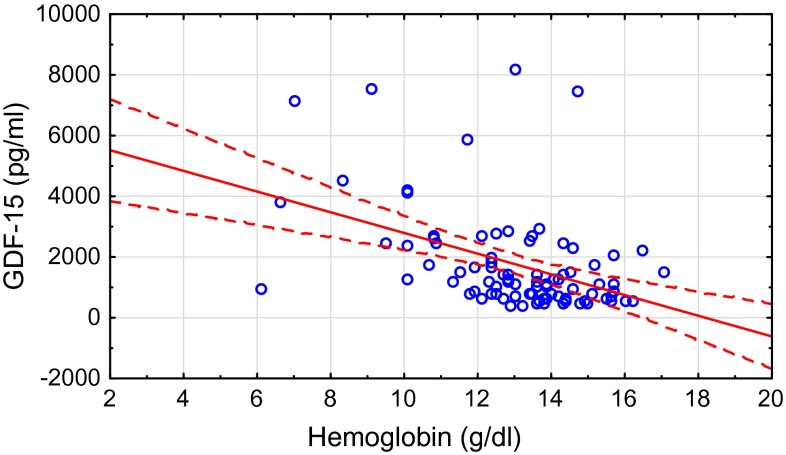
Correlation between GDF-15 and hemoglobin in patients ≥65 years (*r* = −0.52, *P* < 0.005)

**Fig. 2:**
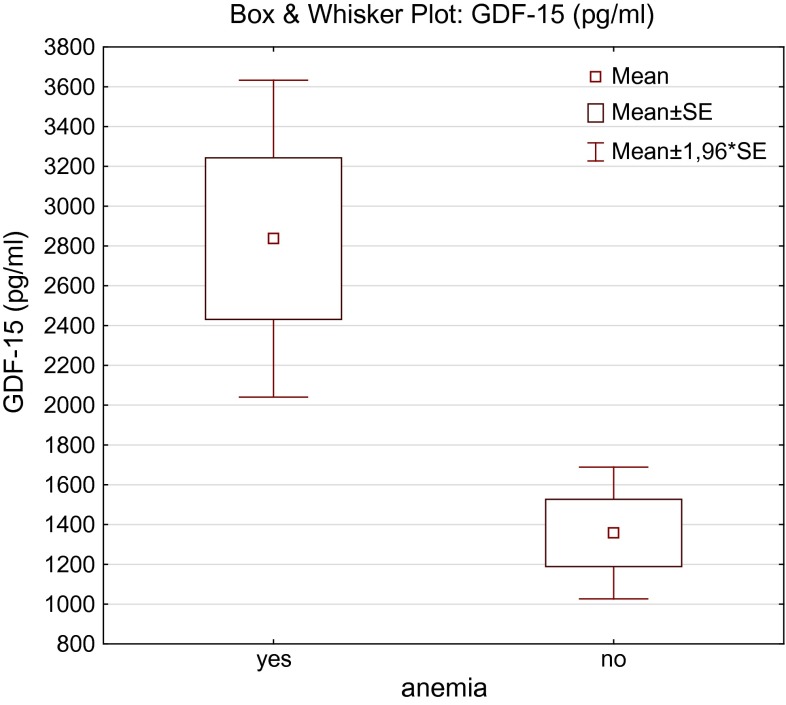
GDF-15 in patients with and without anemia

**Fig. 3:**
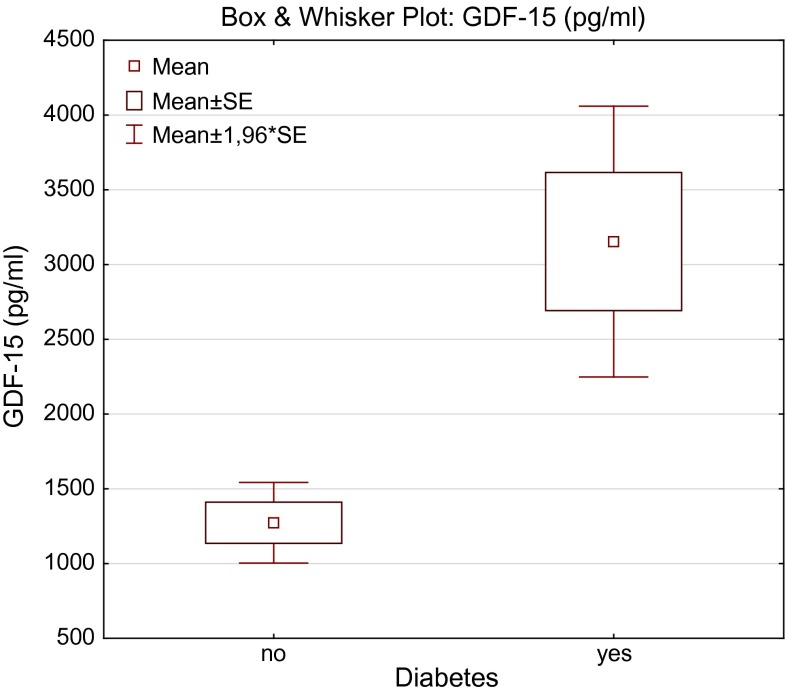
GDF-15 in patients without and with diabetes mellitus

Table 1 presents clinical and laboratory characteristics of the studied population. In patients ≥65 years, serum concentrations of GDF-15 and hsCRP were significantly higher, whereas estimated glomerular filtration was lower. There was no statistically significant difference in hemoglobin, iron, hepcidin, and sTfR concentration between the two studied groups. In univariate analysis serum iron was correlated with IL-6 (*r* = −0.34, *P* < 0.05), hsCRP (*r* = −0.53; *P* < 0.05), and GDF-15 (*r* = −0.38; *P* < 0.05) only in patients ≥65 years. Positive relation between age and GDF-15 (*r* = 0.35; *P* < 0.05) was also observed only in elderly population. In both groups GDF-15 was significantly correlated with hemoglobin (*r* = −0.52 in patients ≥65 years; *r* = −0.46 in patients <65 years; *P* < 0.05) (Fig. 1), eGFR (*r* = −0.5 in patients ≥65 years; *r* = −0.53 in patients <65 years; *P* < 0.05), and inflammatory parameters (Table 2). Hepcidin was significantly correlated with ferritin in both groups (Table 3). GDF-15 was significantly higher in patients with anemia according to WHO definition (Hb less than 12 g/dl in women and 13 g/dl in men) in comparison with their non-anemic counterparts (Fig. 2). In multivariate analysis, hemoglobin was found to be a predictor of logGDF-15 (beta value = 0.36, *P* = 0.006, *R*^2^ = 37 %). We also found significantly higher concentrations of GDF-15 in patients with type 2 diabetes (*n* = 22) in comparison with non-diabetic patients (median 2694 vs. 1020, respectively, *P* < 0.001) (Fig. 3).

**Table 1:** Characteristic of the study population

	<65 years (*n* = 31)	≥65 years (*n* = 56)	Healthy volunteers (*n* = 20)
Age, years	57.5 ± 4.2	77.5 ± 6.8***	51.6 ± 8.8
eGFR, ml/min/1.73 m^2^	70.8 ± 17.4	60.1 ± 19.2**	98.2 ± 15.6^###^
GDF-15 (pg/ml)	679.2 (546.9–956.9)	1451.8 (1055.5–2499.4)**	695.0 (515.8–927.5)
Fe (μg/dl)	79.9 ± 32.9	71.2 ± 38	95.9 ± 25.7
TSAT (%)	27.6 ± 10.8	27.6 ± 14.6	29.0 ± 7.1
Ferritin	160.8 (72.4–227.6)	140.9 (91.6–251.8)	108.9 (61.2–159.1)^#^
Hb (g/dl)	13.2 ± 2	13 ± 2.2	14.0 ± 1.0
hsCRP (mg/l)	3.8 (1.4–16.2)	8.1 (3.8–24.5)*	0.1 (0.1–0.31)^###^
IL-6 (pg/ml)	0.8 (0.2–5)	2.8 (0.5–6.8)	0.1 (0.01–0.35)^###^
sTfR (nmol/l)	17.8 (13.7–25)	18.5 (16.6–23.6)	21.2 (15.5–28.3)
Hepcidin (ng/ml)	33.5 (14.2–61.9)	36.2 (28.2–42.3)	23.3 (19.1–31.8)^#^

* *P* < 0.05, ** *P* < 0.01, *** *P* < 0.001, CKD patients <65 versus >65 years old

^#^ *P* < 0.05, ^###^ *P* < 0.001 healthy volunteers versus CKD patients <65 years

**Table 2:** Spearman’s correlations between hepcidin and classic iron status parameters

	Ferritin	Iron	TSAT
Hepcidin-25			
≥65 years	0.7*	0.01	0.23
<65 years	0.65*	0.39*	0.51*

**Table 3:** Spearman’s correlations between GDF-15 and inflammatory parameters

	hsCRP	IL-6
GDF-15		
≥65 years	0.41*	0.48*
<65 years	0.53*	0.57*

## Discussion

The present report describes iron status in patients with early stages of chronic kidney disease taking into account age differences and inflammation. We found that GDF-15 levels were significantly higher in elderly patients with CKD as well as in patients with anemia. In addition, only in population over 65 years, GDF-15 was negatively correlated with iron levels. In all the patients, strong correlations between inflammatory parameters and iron as well as GDF-15 were observed. In addition GDF-15 was related to kidney function and hemoglobin. In multiple regression analysis, GDF-15 was predicted independently by hemoglobin. However, GDF-15 was not correlated with hepcidin.

GDF-15, also known as macrophage inhibitory cytokine-1 (MIC-1), is implicated in immune and inflammatory response [9]. We found positive correlations between GDF-15 and both IL-6 and hsCRP in our studied population regardless the age. In patients with end-stage renal disease, GDF-15 correlation with hsCRP has also been reported [10]. Interestingly, these associations remained statistically significant only in patients without anemia (regardless the age). In the study conducted by Theurl et al. [11] concerning patients with anemia of chronic disease they also did not find a relation between GDF-15 and either CRP or IL levels. However, in our study GDF-15 levels are significantly increased in patients with anemia that is in line with other research [11, 12]. It may indicate ineffective erythropoiesis and increased erythroid activity [4].

An increase in GDF-15 has been reported in association with faster deterioration in kidney function in patients with type 1 diabetes [13], as well as after acute kidney injury [14]. In the study comprising hemodialysis patients (HD) [15], the authors have shown significantly elevated levels of GDF-15 in HD population, yet no statistically significant relations between GDF-15 and iron status parameters.

In our previous study, BMP-6 and GDF-15, the new players of iron metabolism, were assessed in the population of HD patients. There were no significant differences in BMP6 and GDF-15 levels between patients with and without functional iron deficiency. In this study GDF-15 was related hsCRP and NGAL (neutrophil gelatin associated lipocalin) in patients without functional iron deficiency, while in patients with functional iron deficiency, GDF-15 was related to serum iron and transferrin saturation. Tanno et al. [6] have reported that in patients with thalassemia, serum GDF-15 levels are elevated, which results in the suppression of the iron-regulatory protein hepcidin. Significant induction of GDF-15 has also been observed in individuals with iron deficiency [11, 16]. In our patients with early stages of CKD, GDF-15 correlated with kidney function on only serum iron levels. In our study we did not find these associations that remain in line with HD patients [15].

According to earlier research very high levels of GDF-15 suppressed the expression of hepcidin [6], whereas moderately elevated GDF-15 concentration were positively correlated with hepcidin in kidney allograft recipients [17] and in elderly individuals with anemia of unknown origin [11]. In this study GDF-15 was related to kidney function (creatinine *r* = 0.39, *P* < 0.01, eGFR by MDRD *r* = −0.37, *P* < 0.01), urea (*r* = 0.39, *P* < 0.01), uric acid (*r* = 0.42, *P* < 0.01), hepcidin (*r* = −0.32, *P* < 0.01), IL-6 (*r* = 0.28, *P* < 0.05), hemoglobin (*r* = −0.32, *P* < 0.05), and NGAL (*r* = −0.35, *P* < 0.01). GDF-15 was not related to serum iron, or ferritin. In multivariate analysis, hepcidin was found to be a predictor of GDF-15. Similar to our present study GDF-15 was significantly higher in patients with anemia. In other [18] study on 134 stable heart transplant recipients and 157 patients with chronic heart failure, GDF-15 was significantly higher in patients with anemia compared with non-anemic counterparts in both groups. In univariate analysis in heart transplant recipients, GDF-15 was related to kidney function, age, time after transplantation, hepcidin, sTfR, hemoglobin, transferrin saturation, ejection fraction (EF), and New York Heart Association functional class. GDF-15 was not related to serum iron or ferritin in both groups. In multivariate analysis, sTfR, creatinine, and age were found to be predictors of GDF-15. In univariate analysis in patients with chronic heart failure, GDF-15 was related to creatinine, erythrocyte count, hemoglobin, hepcidin, and total iron-binding capacity and tended to correlate with EF. In multivariate analysis, hepcidin, creatinine, and EF were found to be predictors of GDF-15 in chronic heart failure. In population of CHF, there was no correlation between age and GDF-15 (mean age of CHF patients was 65.31 ± 10.99 years). In addition, GDF-15 was not related to age in kidney allograft recipients (mean age 44.32 ± 12.23 years) [17]. To assess the effect of age on GDF-15, we looked at the possible correlations between age and GDF-15 in healthy volunteers. We found that in healthy subjects GDF-15 was not related to age. Thus in our study higher GDF-15 in the elderly population appears not to be related to age but to lower eGFR and lower hemoglobin. It was hemoglobin, which predicts GDF-15 in 37 % in multivariate analysis.

In another population with subclinical inflammatory status, both hepcidin and GDF-15 levels were increased and showed a positive correlation in anemic patients with type 2 diabetes [19]. In addition, anemic patients with type 2 diabetes without overt renal impairment presented a greater inflammatory state, with increased serum hsCRP, ESR, and IL-6 levels compared with non-anemic counterparts. Lindahl [20] in the elegant review stress the role of GDF-15 as new piece in the puzzle in the world of biomarkers as it has been associated with increased cardiovascular as well as non-cardiovascular mortality, and development and progression of a broad range of diseases, such as coronary artery disease, heart failure, diabetes, cancer, and even cognitive impairment. Similarly Adela and Banerjee [21] in their review paper recognized the relation of high levels of GDF-15 with diabetes, anemia of various etiology, and other comorbidities. Moreover, our work is in line with the previous findings—patients with type 2 diabetes present elevated GDF-15 concentration. It has been shown that GDF-15 may be a predictor of several outcomes in type 2 diabetes [22].

To summarize, as data regarding pathophysiological role of GDF-15 in diabetes, kidney disease and other comorbidities are limited, associations between renal function, anemia and GDF-15 concentrations awaits further studies. Explaining the signaling pathways of GDF-15 may help to understand the exciting puzzle of GDF-15. Normal levels of GDF-15 in the healthy aged were not studied, and the higher values of GDF-15 values found in the present study may possibly be due to age more than CKD levels.
